# Mammary Transcriptome Profile during Peak and Late Lactation Reveals Differentially Expression Genes Related to Inflammation and Immunity in Chinese Holstein

**DOI:** 10.3390/ani10030510

**Published:** 2020-03-19

**Authors:** Ziyin Han, Yongliang Fan, Zhangping Yang, Juan J. Loor, Yi Yang

**Affiliations:** 1 College of Animal Science and Technology, Yangzhou University, Yangzhou 225009, China; mx120170661@yzu.edu.cn (Z.H.); dx120170088@yzu.edu.cn (Y.F.); 2 Joint International Research Laboratory of Agriculture & Agri-Product Safety, Ministry of Education, Yangzhou University, Yangzhou 225009, China; 3 Department of Animal Sciences, University of Illinois, Urbana, IL 61801, USA; 4 Jiangsu Co-innovation Center for the Prevention and Control of Important Animal Infectious Diseases and Zoonoses, Yangzhou University College of Veterinary Medicine, Yangzhou 225009, China

**Keywords:** Chinese Holstein, transcriptome, lactation initiation, mammary gland, differentially expressed genes

## Abstract

**Simple Summary:**

Milk somatic cell count, referring to the total number of somatic cells per milliliter of bovine milk, changes regularly during the lactation cycle. The somatic cell count of healthy cows is usually higher in late lactation than in peak lactation. When the inflammatory response in dairy cow mammary gland becomes more intense, the milk somatic cell count increases together with the reduction of milk quality and yield. Autoimmunity was thought to play an important role in the prevention of mastitis in late lactation of dairy cattle. However, the underlying mechanisms related to the gene expression levels during the process remain unknown. In this study, transcriptome sequencing was performed to screen the differentially expressed genes related to the inflammation and immunity in healthy Chinese Holstein mammary glands. Our findings are helpful to understand the physiological functions of mammary inflammation of Chinese Holstein during late lactation.

**Abstract:**

Somatic cell count (SCC) in milk is widely used in the dairy industry, as an indicator of the health of mammary gland. While the SCC of dairy cattle was higher in late lactation than in peak lactation, its association with gene expressions of mammary gland were largely unknown. In this study, a transcriptomic sequencing approach and bioinformatics analysis were used to investigate the differential expressed genes (DEGs) associated with inflammation and immunity between peak and late periods of lactation in Chinese Holstein. A total of 446 DEGs (padj < 0.05 and fold change >2) were identified, 50 of which belonged to seven pathways and five terms related to inflammation and immunity. Our data suggested that the activation of nuclear transcription factor-κB (NF-κB) pathway and Toll-like receptor signaling pathway caused inflammatory response, and the activation of chemokine signaling pathway and cytokine–cytokine receptor interaction signaling pathway caused a protective immune response to ensure dairy cows health during late lactation. Our findings deepen the understanding of the molecular mechanism and physiological functions of mammary inflammation in Chinese Holstein during late lactation.

## 1. Introduction

The somatic cell count (SCC) referred to as the total number of somatic cells per milliliter of milk is widely used to measure the health status of dairy cows and the quality of milk [1]. In general, the quality and yield of milk was negatively associated with SCC [2], which is an important indicator of clinical and subclinical bovine mastitis. The United States stipulates that SCC in bovine milk must be less than 750,000/mL, while the European Union is stricter, which adheres to SCC in bovine milk must be less than 400,000/mL, and China’s standard is that SCC in bovine milk must be less than 500,000/mL [3,4].

Previous studies have focused on the interfering factors of SCC and its association with the quality and yield of milk [2,5,6]. Several studies have demonstrated that the SCC was higher in late lactation than that in peak lactation [7,8,9,10,11]. Moreover, the autoimmune reaction and the innate immune response of dairy cattle protect them from mastitis during lactation, especially in the late stage [12], the changes in gene expression level in mammary gland tissue at different stages of lactation are largely unknown. In recent years, transcriptome technology was used to detect changes in dairy cow gene expression level, however, most sampling methods cannot eliminate individual differences [13,14].

The purpose of this study is to screen differential expression genes (DEGs) related to inflammation and immunity in healthy Chinese Holstein during the peak and late lactation period by transcriptome sequencing without slaughter.

## 2. Materials and Methods

### 2.1. Ethics Statement

This study was performed in strict accordance with the Regulations of the Administration of Affairs Concerning Experimental Animals (Ministry of Science and Technology, China, revised in 2004) and approved by the Institutional Animal Care and Use Committee (IACUC) of the Yangzhou University Animal Experiments Ethics Committee (Permit Number: SYXK (Su) IACUC 2012-0029). All animals were reared in compliance with national regulations and according to procedures approved by the veterinary services of China.

### 2.2. Animals Selection and Samples Collection

A total of 33 healthy Chinese Holstein used in this study were selected from the experimental farm of Yangzhou University. All the animals with similar body weights (628.33 ± 20.05 kg) in the second lactation period did not have a history of mastitis, and fed with total mixed ration (TMR), including 23% alfalfa hay and 7% Chinese wild rye hay with a forage-to-concentrate ratio of 45:55 [15]. The milk yield of each individual was recorded twice per day. Milk samples were collected into 5 mL (for bacterial isolation and identification) and 50 mL (for the determination of SCC) from the left anterior mammary region on the 90th, 150th, 210th, and 270th day of lactation and transported on ice to the lab within 2 h. Three Chinese Holstein (A, B, C) were randomly selected to obtain a biopsy: 1−2 g mammary gland tissue in the same quarter that was providing the milk sample by surgical methods in vivo on the 90th and 270th day of lactation [16,17]. The skin of the selected biopsy site was shaved and disinfected with ethanol (75%), then anaesthetized with SU-MIAN-XIN (846 compound anesthetic agent, 35 mg, intravenously) and injected subcutaneously with 1 mL of procaine. A 1.5 cm incision was made at the midpoint of the selected quarter. The connective tissue was blunt-dissected away to expose the mammary parenchymal tissue using disinfectant shears and tweezers. The mammary tissues biopsy (1–2 g) was obtained and washed with diethylpyrocarbonate (DEPC)-treated double-distilled water. Then, mammary tissues were immediately frozen in liquid nitrogen until RNA isolation. While obtaining mammary tissue samples, 11-mm Michel wound clips (#9534503, Henry Stein, Inc., Melville, NY, USA) were used to close the skin incision. Then, the skin incision was covered with iodine ointment (#1048023, Povidone Iodine Ointment, Guangdong qingfa pharmaceutical co. LTD, Guangzhou, China).

### 2.3. Microbiological Study

Upon arrival, bacterial isolation was performed as described with minor modification [18]. In brief, 2 mL of milk was diluted into 2 mL of phosphate buffer saline (PBS) and 100 μL of diluent was plated onto a blood agar and a MacConkey plate, and incubated at 37 °C aerobically for 24–48 h. Based on the morphology of colonies, one of identical colony of each sample was expended cultured in nutrient broth at 37 °C. After 24–48 h incubation, 200 μL of culture was used for DNA extraction with the Roche High Pure PCR Template Preparation Kit (Roche Diagnostics GmbH, Mannheim, Germany). Extracted DNA was subjected to a PCR assay with a broad-range PCR primer targeting the 16S rDNA gene of pathogenic and nonpathogenic bacteria [19]: Forward primer = 5′- AGAGTTTGATCCTGGCTCAG -3′; reverse primer = 5′- TACGGCTACCTTGTTACGACT -3′. The amplicons were sequenced (Genscript, Nanjing, China) and the BLASTn was performed to determine the bacterial species.

### 2.4. Determination and Analysis of SCC in Milk Samples

The 20-mL milk samples with 0.015 g of potassium dichromate were sent to Nanjing Agricultural University to determine SCC (Agricultural Product Safety Testing Center of Nanjing Agricultural University, Jiangsu Province, China). Further analysis was performed with SCS calculated with SCC as described [20].

### 2.5. Total RNA Extraction and cDNA Library Construction

Total RNA was extracted using the mirVana™ miRNA Isolation Kit (Ambion-1561) following the manufacturer’s protocol. RNA integrity was evaluated using the Agilent 2100 Bioanalyzer (Agilent Technologies, Santa Clara, CA, USA). The samples with RNA Integrity Number (RIN) ≥ 7 were subjected to the subsequent analysis.

The libraries were constructed using TruSeq Stranded mRNA LT Sample Prep Kit (Illumina, San Diego, CA, USA, RS-122-2101) according to the manufacturer’s instructions. Then, these libraries were sequenced on the Illumina sequencing platform (HiSeq™ 2500) and 125 bp paired-end reads were generated.

Raw reads were processed using NGS QC Toolkit [20,21]. The reads containing ploy-N and the low-quality reads were removed to obtain the clean reads. Then, the clean reads were mapped to reference bovine genome UMD3.1 using Bowtie2 2.3.5.1 [21,22] and TopHat 2.1.1 [22,23].

### 2.6. Gene Expression Level Analysis

Gene expression was calculated using the FPKM method, which is the number of fragments per kilobase length from a gene in each million fragments [24]. The read counts of each gene were obtained by HtSeq-count 0.9.1 [25]. PCA analysis was performed using the gene expression profiles. Genes were divided into high (≥500 FPKM), medium (≥10 to 500 FPKM), and low expression (<10 FPKM) [26]. DEGs were identified using the DESeq R package (1.18.0) (2012) functions estimate size factors and nbinom test [27]. False discovery rate (FDR, padj) <0.05 and fold change >2 was set as the threshold for DEGs.

### 2.7. Functional Annotation and Pathway Analysis of DEGs

Hierarchical cluster analysis of DEGs was performed to explore transcripts expression pattern. DAVID 6.8 (https://david.ncifcrf.gov/) [28] were used for GO (gene ontology) annotation analyses of DEGs. KEGG pathways analyses of DEGs were implemented by KOBAS 3.0 online program (http://kobas.cbi.pku.edu.cn/index.php) [29]. GO enrichment and KEGG pathway enrichment analysis of DEGs were, respectively, performed using R based on the hypergeometric distribution. The calculation for this was formula (1), where N is the number of genes with a pathway annotation in all genes; n is the number of differentially expressed genes in N; M is the number of genes annotated as a particular pathway in all genes; and m is the number of differentially expressed genes annotated as a particular pathway. GO terms and KEGG pathways with padj < 0.05 were significantly enriched in DEGs.

(1) $$p=1-\overset{m-1}{\underset{i=0}{\sum }}\frac{\left(\begin{array}{c} M\\ i \end{array}\right)\left(\begin{array}{c} N-M\\ n-i \end{array}\right)}{\left(\begin{array}{c} N\\ n \end{array}\right)}$$

### 2.8. PPI Network Construction and Analysis

A protein–protein interaction (PPI) network was created using Cytoscape v3.7.2 to further understand and predict the biological activity of the identified DEGs related to inflammation and immunity based on GO and KEGG enrichment analysis [30]. The DEGs’ encoding proteins and their interacting partners were computed from the String v11.0 database for PPI network construction [31]. This PPI network was subsequently visualized in Cytoscape.

### 2.9. Validation of Sequencing Data by qRT-PCR

Ten DEGs were selected from DEGs at random to validate the transcriptome sequencing results. The primers (Table 1) used for quantification in the study were designed using Primer-BLAST on the NCBI website. In all cases, primers designed for quantitative real-time PCR (qRT-PCR) spanned exon–exon boundaries. In the study, ribosomal protein S9 (*RPS9*) [32] and *β-actin* were used as the housekeeping gene. qRT-PCR was performed using the Light Cycler^®^ 480 System (Roche, Hercules, CA, USA) with SYBR Green PCR Master Mix (TaKaRa SYBR^®^ PrimeScript™ RT-PCR Kit, Dalian, China) according to the manufacturer’s instructions (n = 9 experiments, three replicates per experiment). Relative expression was calculated using the 2^−ΔΔCt^ method [33]. qRT-PCR response procedures for: 40 cycles of 95 °C for 30 s, 95 °C for 10 s, 60 °C for 30 s.

### 2.10. Statistical Analysis

All the statistical analyses were performed with Software Package for Social Sciences (SPSS) Version 19.0 (IBM, Among, New York, NY, USA). The differences in SCS of milk collected during different days of lactation was compared with one-way analysis of variance (ANOVA) with comparison among means made by Duncan’s multiple range test. The day of lactation was set as the X (independent) variable and the SCS in milk was set as the Y (outcome) variable. The relative expressions of mRNA were analyzed by using independent sample T-test and mean plots ± 95% confidence intervals. The Pearson correlation coefficient analysis were performed to compare the data obtained from transcriptome sequencing and qRT-PCR. Each biological repetition was carried out involving three replicates. All data were presented as the mean ± standard error (SE) and considered statistically significant when *p* < 0.05.

## 3. Results

### 3.1. Microbiological Analysis

Among the milk samples from enrolled cows, 91.67% (22/24) of them were positive for culturing, whereas 8.33% (2/24) of them yielded no growth. The sterile growth was from the milk on the 90th and 270th day of lactation of the same individual. One bacterial specie, *Escherichia coli* was identified in the positive cultures.

### 3.2. Daily Milk Yield and SCC in Milk Samples

Daily milk yield, SCC, and SCS were shown in Table 2. The daily milk yield of all the tested animals showed a continuous decline during the trial period. SCC and SCS of Chinese Holstein showed an increasing trend during the test period. Compared with the 90th day of lactation, the SCS of the 33 animals increased significantly on the 270th day of lactation.

### 3.3. Analysis of cDNA Libraries

Transcriptome sequencing results and quality parameters were shown in Table 3. After the quality control of sequencing data, the number of clean bases accounted for more than 98.05% of the raw bases. The sequences with Q30 and above accounted for more than 95.92%. The GC content reached between 47.50% and 49.00%. The results of comparison between sequencing data and genome information were shown in Table 4. The total reads of each sample were more than 90.90% compatible with the bovine reference genome.

### 3.4. Gene Expression in Different Samples

Two clusters were found: Peak lactation and late lactation (Figure S1). The same genes from different dairy cows in same stages could be classified into the same clusters, indicating that the main distinctions in the mRNA expression profiles occurred in the different stage. Total expressed genes are classified into high (≥500 FPKM), medium (≥10 to 500 FPKM), and low (<10 FPKM) expression (Table 5).

### 3.5. Screening of Differentially Expressed Genes

**Figure 1:**
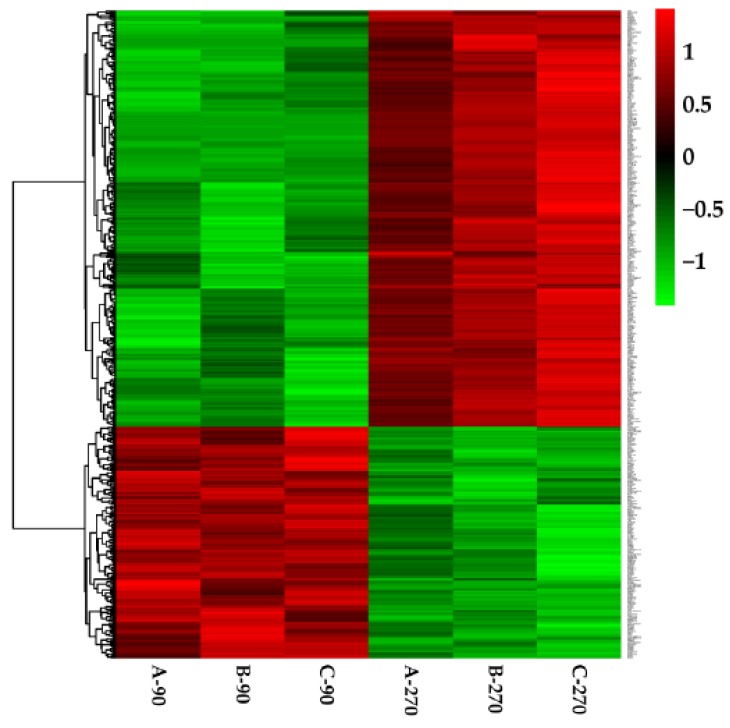
Heat map of the differentially expressed genes. Green indicates lower expression genes and red indicates higher expression genes.

**Figure 2:**
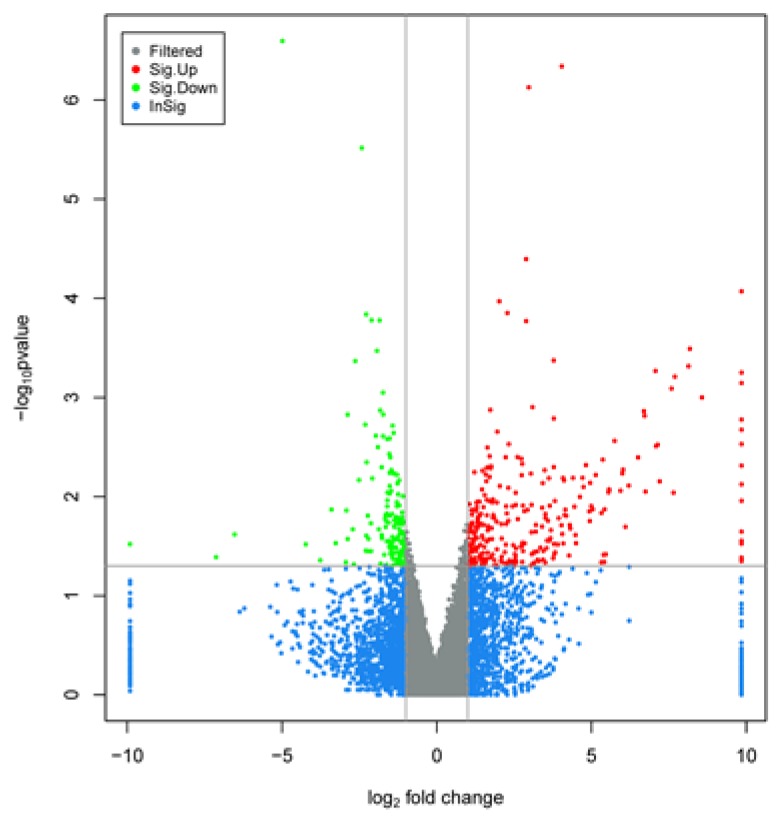
Volcano plot displaying differential expressed genes in bovine mammary tissues during peak (A-90, B-90, C-90) and late (A-270, B-270, C-270) lactation. The y-axis corresponded to the mean expression value of log_10_(p-value), and the x-axis displayed the log_2_ fold change value. The red and green dots represented the significant differentially expressed gene (padj < 0.05) in bovine mammary tissue during peak and late lactation; the blue and grey dots represented the transcripts whose expression levels did not reach statistical significance in bovine mammary tissue during peak and late lactation.

There were 291 upregulated genes and 155 downregulated genes were identified during late lactation in bovine mammary tissues, compared to peak lactation (Figure 1 and Figure 2).

### 3.6. GO and KEGG Enrichment Analysis of DEGs

**Figure 3:**
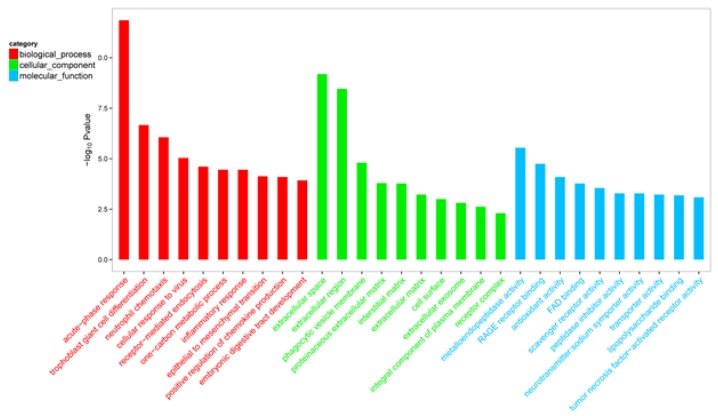
Gene ontology functional enrichment analysis of differentially expressed genes. Only top 10 significant biological process, cellular component, and molecular function terms were listed, respectively.

DEGs were classified by GO enrichment according to biological process, cellular component (GO-CC), and molecular function (GO-MF). The top 10 terms of GO-BP, GO-CC, and GO-MF were shown in Figure 3, respectively. Among significantly enriched (padj < 0.05) top 10 GO-BP terms, there were five GO-BP terms related to inflammation and immune, including 30 genes (Table 6).

**Figure 4:**
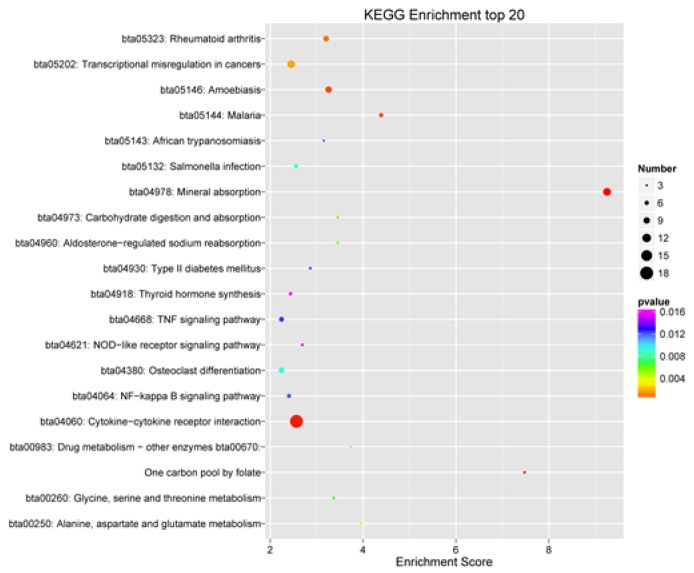
Top 20 significant pathways of Kyoto Encyclopedia of Genes and Genomes enrichment analysis of differentially expressed genes.

KEGG enrichment analysis of DEGs revealed 43 significantly enriched pathways. The top 20 significantly enriched pathways (padj < 0.05) were listed in Figure 4. Seven of the 43 significantly enriched pathways (padj < 0.05) were associated with inflammation and immune response, including 33 genes (Table 7).

### 3.7. PPI Network Analysis

**Figure 5:**
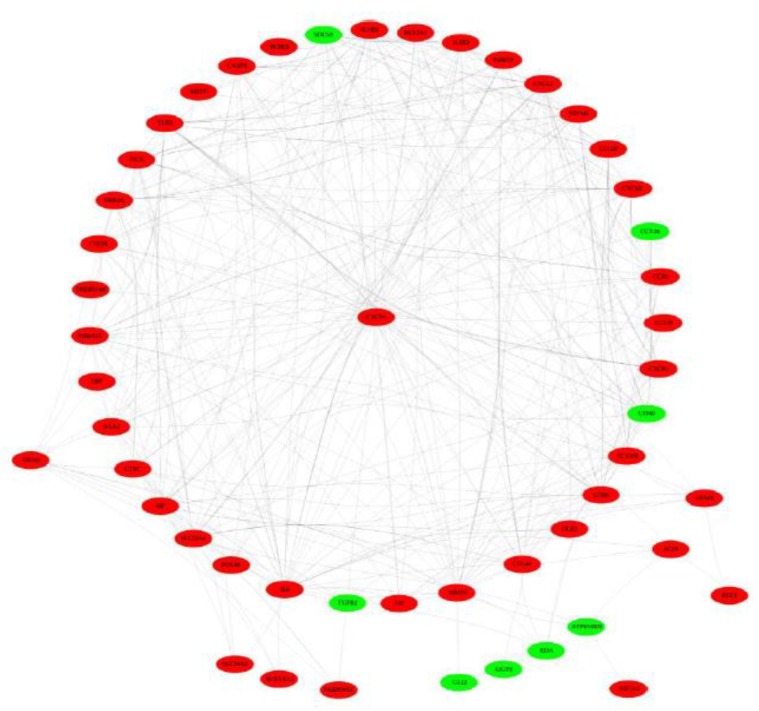
A protein–protein interaction network of differentially expressed genes related to inflammation and immunity. The node in red and green, respectively indicated that the gene was upregulated and downregulated in on late lactation compared with peak lactation.

The PPI network of DEGs related to inflammation and immunity (Figure 5) revealed the biological activity and interactive relationship of their encoding proteins.

### 3.8. Verification Results of qRT-PCR

**Figure 6:**
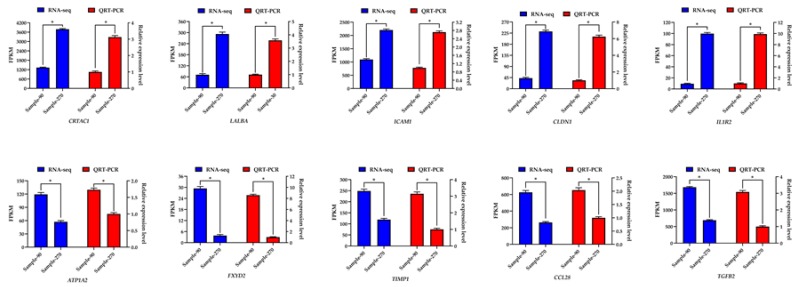
Expression level of ten differentially expressed genes detected by qRT-PCR using *β-actin* as reference gene and RNA-Seq. “*”: *p* < 0.05.

The results showed that the genes expression trends were consistent between sequencing data and qRT-PCR results. Moreover, the correlation coefficient of the sequencing data and qRT-PCR result using *β-actin* as reference gene reached 0.992 (R^2^ = 0.984) (Figure 6), which was highly significant (*p* < 0.000). The correlation coefficient of the sequencing data and qRT-PCR result using *RPS9* as reference gene reached 0.981 (R^2^ = 0.962) (Figure S2), which was highly significant (*p* < 0.000) too. The reliability of the sequencing data was high.

## 4. Discussion

The SCS of milk at 270th day of lactation was significantly higher than that at 90th day of lactation. There were five GO-BP terms and seven pathways (padj < 0.05) related to inflammation and immune response. The innate immune system of bovine mammary gland is composed of teat duct, body fluid, and immune cells [34]. The teat duct is closed in nonlactation, scilicet, a physical barrier separates pathogens from bovine mammary gland. At the late stage of lactation, with the increasing times of milking, the pathogens can easily enter the bovine mammary gland due to the mechanical injury to the nipples of cow [35]. Therefore, some pathogens enter bovine mammary gland and the SCC of milk in late lactation was higher than that in peak lactation. In this study, the expression level of Toll-like receptor 2 (*TLR2*) was significantly upregulated in order to receive more signals released by intruders to activate the Toll-like signaling pathway, inflammatory response term, and positive regulation of chemokine production term. The activated *TLR2* induced the activation of MYD88 innate immune signal transduction adaptor (*MyD88*) [36,37], which can activate the NF-κB signaling pathway to produce nuclear transcription signaling, cytokines, and chemokines [38]. From that moment, the chemokine signaling pathway and the cytokine–cytokine receptor interaction were activated. Cytokines such as tumor necrosis factor alpha (*TNF-α*) and interleukin 1 beta (*IL-1β*) were responsible for amplifying signals, while C-X-C motif chemokine ligand 8 (*CXCL8*) was bound to its specific receptor chemokine (C-X-C motif) receptor 1 (*CXCR1*) and C-X-C motif chemokine receptor 2 (*CXCR2*). Neutrophils rapidly respond to chemotactic signals and migrate to inflammatory sites to kill pathogens [39]. Meanwhile, the neutrophil chemotaxis term was activated. Compared with peak lactation, the expression levels of *CXCL8*, *CXCR1*, *CXCR2*, *TNF-α,* and *IL-1β* were upregulated at late lactation. These results were consistent with Gilbert et al. [38].

The study of Gilbert et al. [38] showed that *E. coli* crude lipopolysaccharide (LPS) preparation stimulated *TLR2* and Toll-like receptor 4 (*TLR4*) in bovine mammary epithelial cells. In this study, there was no significant difference in the expression levels of *TLR4*. We found that there was also no significant difference in the expression levels of the potential downstream genes of *TLR4*: Interferon regulatory factor 3 (*IRF3*), C-X-C motif chemokine ligand 10 (*CXCL10*), chemokine (C-C motif) ligand 2 (*CCL2*), C-C motif chemokine ligand 5 (*CCL5*). The analysis of DEGs associated with inflammation and immunity indicated that the analyzed cows have a localized inflammatory reaction, which was consistent with the determination of SCC. In addition, Tahir Usman et al. [40] found that interleukin 17F (*IL-17F*) and interleukin 17A (*IL-17A*) could be powerful candidate genes of mastitis resistance and the significant single nucleotide polymorphisms (SNPs) might be useful genetic markers against mastitis in both dairy and dual-purpose cattle. In this study, the expression levels of *IL-17F* and *IL-17A* did not change significantly.

Zinc finger protein A20 (*A20*), encoded by TNF-α induced protein 3 (*TNFAIP3*), is a deubiquitinase that can be induced by *TNF-α* and *IL-1β* and then transcribed rapidly [36,37,41,42]. Ubiquitin is activated by the ubiquitin-activating enzymes (E1) in the presence of ATP and binds to E1. The activated ubiquitin molecule then transfers to the ubiquitin binding enzyme (E2). Ubiquitin ligases (E3) attract ubiquitin-E2 complexes and substrate proteins, and ubiquitin is transferred from E2 to the substrate. Substrate-ubiquitin complexes are removed by deubiquitinating enzyme B [38,43]. In this study, the expression level of *TNFAIP3* was significantly higher at late lactation compared with peak, namely A20 acted on deubiquitinated proteins such as tumor necrosis factor receptor-associated protein 6 (*TRAF6*) and receptor-interacting protein 1 (*RIP1*) in the NF-κB signaling pathway, thereby negatively regulating the NF-κB signaling pathway, inhibiting the production of inflammatory signals, and ultimately reducing the damage caused by excessive inflammation [44]. The results showed that there was no significant difference in the expression levels of *TRAF6* and *RIP1* during the two test periods, indicating that the activation and inhibition of NF-κB signaling pathway belonged to the category of innate immune of bovine mammary gland and did not lead to mastitis and autoimmune diseases of the analyzed cows.

Solute carrier family 11 member 1 (*SLC11A1*) is known as natural resistance related macrophage protein. *SLC11A1* is mainly distributed in the mammalian reticuloendothelial system, especially in macrophage phagocytic lysosome membrane. When pathogens invade cells, they activate pattern-recognition receptors, such as Toll-like receptors [45]. Endosomes are formed after the pathogens endocytosis by macrophages. *SLC11A1*, significantly upregulated at the late lactation, transported the metal ions necessary for the survival of pathogens out of the endosome, thus killing the pathogens [46]. Studies on dairy cows have shown that *SLC11A1* is resistant to a variety of pathogens [47,48,49,50]. Joo et al. have proved that the mRNA expression of *SLC11A1* in resistant dairy cows was significantly higher than that in susceptible dairy cows. Mastitis resistant dairy cows can be selected according to the difference in *SLC11A1* expression [51].

## 5. Conclusions

In this study, a total of 446 DEGs were identified in the mammary tissue of late lactation (high SCC period) and peak lactation (low SCC period) of Chinese Holstein. Functional analysis showed that 50 DEGs related to immunity and inflammation such as *TLR2, TNF-α, IL-1, CXCR1, CXCR2, CXCL8, TNFAIP3, TRAF6, RIP1,* and *SLC11A1*. Further studies are warranted to further explore the molecular mechanism of inflammation and immune response regarding to these DEGs.

## Figures and Tables

**Table 1:** Primers used in quantitative real-time PCR.

Gene	Forward Primers (5′-3′)	Reverse Primers (5′-3′)	Length (bp)	GenBank ID
*SLC11A2*	AGTTGACCTCCCTGGACATCT	CACGTTCGGAGGAACACTGG	132	NM_001101103.1
*CD40*	GAACAACACGTGGGGACGAA	CCGCTTCTTGGTTATGTTCCTG	147	NM_001105611.2
*ICAM1*	GGAGGTGCCGGAATATCAAT	GGCCCACTTCCTCCTTGATTA	139	NM_174348.2
*CCR1*	TCCGACTCACTCAGGACCTT	CCACGGGTCAAGGGAAATGT	146	NM_001077839.1
*IL1R2*	ACTGAAGGTGAAAGGCCTGG	CGAAGGTGGACACACCCATT	150	NM_001046210.2
*ATP1A2*	AGCTGTGGTCATCGTCACTG	TCCGCGTTGATCTGCATCTT	138	NM_001081524.1
*FXYD2*	TATGGACAGGTGGTACCTGGG	CAGCGGAATCTTTTGCTGAGG	150	NM_174320.4
*SLC30A1*	TCACGCTACCACCATTCAGC	TTTCCAGACTGGGCTTGTGG	135	NM_001205893.2
*CCL28*	AAGCAGCCAAGAAAGAGGCT	CCTCTGTGCAGCTTCATCTGT	150	NM_001101163.1
*TGFB2*	ACCCTCGGAAAATGCCATCC	GCACTCTGGCTTTTGGGTTC	149	NM_001113252.1
*RPS9*	CCTCGACCAAGAGCTGAAG	CCTCCAGACCTCACGTTTGTTC	62	NM_001034034.2
*β-actin*	CATCCTGACCCTCAAGTA	CTCGTTGTAGAAGGTGTG	91	NM_173979.3

**Table 2:** Milk yield, somatic cell count, somatic cell score in different test days (means ± SE).

Test Days	90	150	210	270
Daily milk yield (Kg)	34.40 ± 0.05 ^a^	33.17 ± 0.04 ^b^	29.62 ± 0.04 ^c^	26.51 ± 0.04 ^d^
Somatic cell count (SCC) (10^4^)	24.03	24.00	32.00	46.98
Somatic cell score (SCS)	4.26 ± 0.01 ^c^	4.26 ± 0.01 ^c^	4.68 ± 0.01 ^b^	5.23 ± 0.01 ^a^

Note: Different letters a, b, c in the same row differ significantly (*p* < 0.05) by Duncan’s test. The numbers in line 4 reflected a mean.

**Table 3:** Basic information of sequencing reads and bases.

Sample	Raw Reads	Raw Bases	Clean Reads	Clean Bases	Valid Ratio (Base)	Q30	GC
A-90	61,255,240	7.66 Gb	60,490,684	7.56 Gb	98.72%	97.14%	48.50%
B-90	61,664,866	7.71 Gb	60,994,408	7.62 Gb	98.89%	97.33%	47.50%
C-90	59,050,772	7.38 Gb	58,314,034	7.29 Gb	98.72%	97.11%	49.00%
A-270	71,589,742	8.95 Gb	70,550,840	8.82 Gb	98.53%	96.48%	48.50%
B-270	77,932,606	9.74 Gb	76,857,188	9.61 Gb	98.60%	96.44%	49.00%
C-270	64,104,356	8.01 Gb	62,867,970	7.86 Gb	98.05%	95.92%	49.00%

**Table 4:** Statistics of total reads and mapped reads.

Item	A-90	B-90	C-90	A-270	B-270	C-270
Total reads	60,490,684	60,994,408	58,314,034	70,550,840	76,857,188	62,867,970
Total mapped	54,988,154 (90.90%)	56,242,133 (92.21%)	54,192,238 (92.93%)	64,298,141 (91.14%)	69,765,025 (90.77%)	58,484,736 (93.03%)

**Table 5:** Statistics of gene expression in samples.

Gene Expression	A-90	B-90	C-90	A-270	B-270	C-270
High expression genes (≥500 FPKM)	82	61	81	63	79	89
Medium expression genes (≥10 to 500 FPKM)	4294	3207	5687	3709	4947	5641
Low expression genes (<10 FPKM)	11,585	12,311	10,692	11,962	11,404	10,490
Nonexpressed genes	5535	5917	5036	5542	4846	5056
Total expressed genes	15,961	15,579	16,460	15,734	16,430	16,220

**Table 6:** Significantly enriched gene ontology (GO) terms related to inflammation and immunity.

Term ID	Term	padj	Gene Name	Number of Genes
GO:0006953	Acute-phase response	<0.001	*M-SAA3.2*; *ORM1*; *SERPINF2*; *SAA3*; *LBP*; *IL6*; *CD163*; *HP*; *IL1RN*	18
GO:0030593	Neutrophil chemotaxis	<0.001	*PDE4B*; *CCL19*; *CCL20*; *S100A8*; *S100A9*; *CXCL8*; *CSF3R*; *TREM1*	8
GO:0098586	Cellular response to virus	<0.001	*IKBKE*; *CCL19*; ***GLI2***	6
GO:0006954	Inflammatory response	< 0.001	*RELT*; *S100A12*; *TNFRSF6B*; *OLR1*; *CCL19*; *CCL20*; *CCR1*; *MEFV*; *CASP4*; *SLC11A1*; *CXCL8*; ***GGT5***; ***CD40***; *TLR2*	7
GO:0032722	Positive regulation of chemokine production	<0.001	*LBP*; *IL6*; *TLR2*	9

Note: The gene in bold indicated that the gene was downregulated on the 270th day of lactation. Other genes were upregulated on the 270th day of lactation.

**Table 7:** Significantly enriched pathways related to inflammation and immunity.

KEGG-Pathway	Signal Path	padj	Gene Name	Number of Genes
bta04060	Cytokine–cytokine receptor interaction	<0.001	*CCL19; CCL20; **CCL28**; CCR1; CD40; CSF3R; CXCL2; CXCL8; CXCR1; CXCR2; **EDA**; IL1R2; IL6; LIF; OSMR; RELT; **TGFB2**; TNFRSF6B*	18
bta05323	Rheumatoid arthritis	0.001	*ACP5; **ATP6V0D2**; CCL20; CXCL8; ICAM1; IL6; **TGFB2**; TLR2*	8
bta04064	NF-kappa B signaling pathway	0.012	*BCL2A1; CCL19; CD40; CXCL8; ICAM1; LBP*	6
bta04668	TNF signaling pathway	0.013	*CCL20; CXCL2; ICAM1; IL6; LIF; MMP9; **SOCS3***	7
bta04620	Toll-like receptor signaling pathway	0.019	*CD40; CXCL8; IKBKE; IL6; LBP; TLR2*	6
bta04142	Lysosome	0.024	*ABCA2; ACP5; ATP6V0D2; CD68; CTSC; SLC11A1; SLC11A2*	7
bta04062	Chemokine signaling pathway	0.035	*CCL19; CCL20; **CCL28**; CCR1; CXCL2; CXCL8; CXCR1; CXCR2; HCK*	9

Note: The gene name in bold indicated that the gene was downregulated on the 270th day of lactation. Other genes were upregulated on the 270th day of lactation.

## Supplementary Materials

The following are available online at https://www.mdpi.com/2076-2615/10/3/510/s1, Figure S1: PCA analysis of the mRNA of genes. Figure S2: Expression level of ten differentially expressed genes detected by qRT-PCR using RPS9 as reference gene and RNA-Seq.
