# Supplementary figures and images for: Mammary Transcriptome Profile during Peak and Late Lactation Reveals Differentially Expression Genes Related to Inflammation and Immunity in Chinese Holstein
Source: Animals (Basel). 2020 Mar 19;10(3):510. doi: 10.3390/ani10030510 (PMC7143190; doi:10.3390/ani10030510)

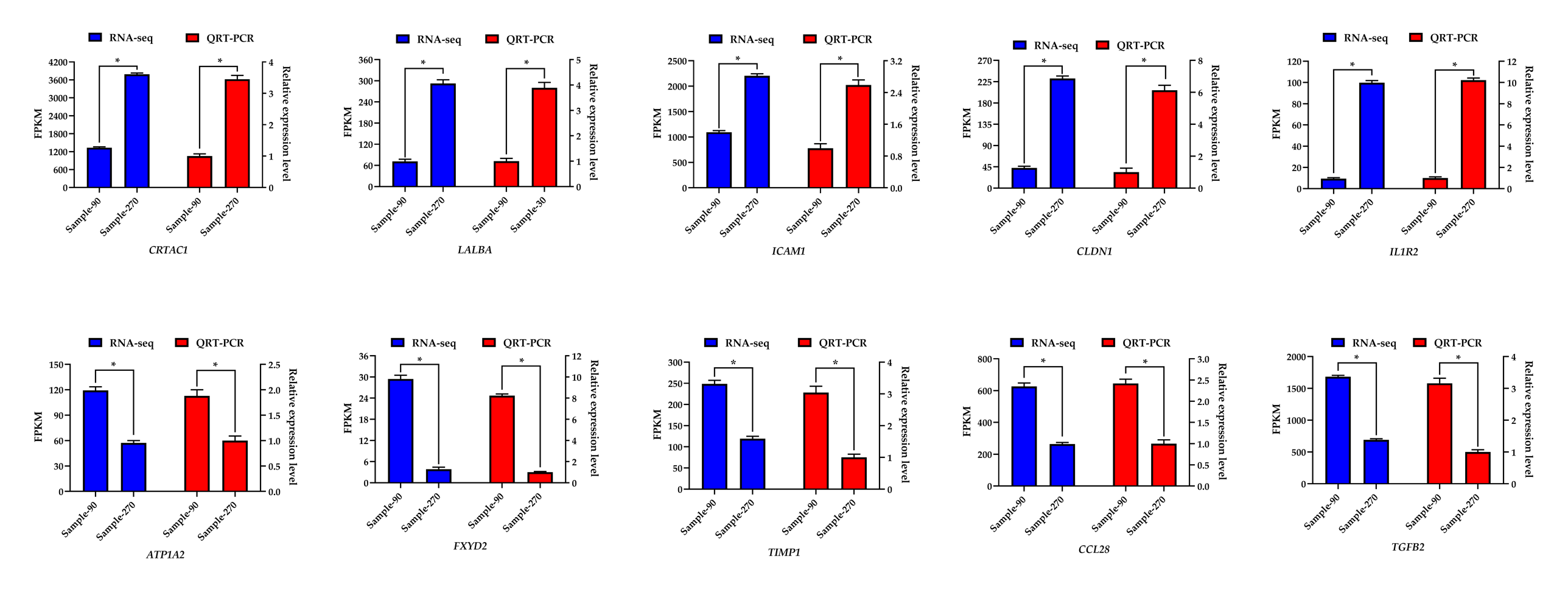

Supplement: Supplementary file 1 [file animals-10-00510-s001.zip › animals-722839-Supplementary Materials/Figure S2 Expression level of ten differentially expressed genes detected by qRT-PCR using RPS9 as reference gene and RNA-Seq..png]
